# Impairment in novelty-promoted memory via behavioral tagging and capture before apparent memory loss in a knock-in model of Alzheimer’s disease

**DOI:** 10.1038/s41598-022-26113-1

**Published:** 2022-12-24

**Authors:** Tabitha Broadbelt, Menekse Mutlu-Smith, Daniel Carnicero-Senabre, Takaomi C. Saido, Takashi Saito, Szu-Han Wang

**Affiliations:** 1 Centre for Clinical Brain Sciences, The University of Edinburgh, Chancellor’s Building, 49 Little France Crescent, Edinburgh, EH16 4SB UK; 2 Laboratory for Proteolytic Neuroscience, RIKEN Brain Science Institute, Saitama, 351-0198 Japan; 3 Department of Neurocognitive Science, Institute of Brain Science, Nagoya City University Graduate School of Medical Sciences, Aichi, 467-8601 Japan; 4 Present Address: Centro de Investigación Biomédica en Red Sobre Enfermedades Neurodegenerativas (CIBERNED), Instituto de Investigación Sanitaria La Paz (IdiPaz), Department of Biochemistry and Instituto de Investigaciones Biomédicas Alberto Sols UAM-CSIC, Faculty of Medicine, Autonomous University of Madrid, Madrid, Spain

**Keywords:** Long-term memory, Short-term memory, Spatial memory, Learning and memory, Alzheimer's disease

## Abstract

Alzheimer’s disease (AD) is associated with cognitive impairments and age-dependent memory deficits which have been studied using genetic models of AD. Whether the processes for modulating memory persistence are more vulnerable to the influence of amyloid pathology than the encoding and consolidation of the memory remains unclear. Here, we investigated whether early amyloid pathology would affect peri-learning novelty in promoting memory, through a process called behavioral tagging and capture (BTC). *App*^*NL-G-F/NL-G-F*^ mice and wild-type littermates were trained in an appetitive delayed matching-to-place (ADMP) task which allows for the assessment of peri-learning novelty in facilitating memory. The results show that novelty enabled intermediate-term memory in wild-type mice, but not in *App*^*NL-G-F/NL-G-F*^ mice in adulthood. This effect preceded spatial memory impairment in the ADMP task seen in middle age. Other memory tests in the Barnes maze, Y-maze, novel object or location recognition tasks remained intact. Together, memory modulation through BTC is impaired before apparent deficits in learning and memory. Relevant biological mechanisms underlying BTC and the implication in AD are discussed.

## Introduction

The number of people suffering from Alzheimer’s disease (AD) is estimated to rise to 152.8 million by 2050^1^. Currently, AD is clinically diagnosed with cognitive impairments and the distinct neuropathological features of the disease. Earlier detection of the disease will be beneficial to the development of therapeutic avenues. Genetic mouse models were developed to understand the amyloid pathology and the cognitive deficits in AD^2^. Saito et al*.* used a targeted knock-in approach to insert 3 familial AD mutations into the humanised murine APP gene to generate the *App*^*NL-G-F/NL-G-F*^ mouse line that selectively expresses increased Aβ40 and Aβ42^3^. In homozygous *App*^*NL-G-F/NL-G-F*^ mice, amyloid deposition in the cortex begins around 3 months after birth reaching its peak around 6–9 months^3,4^. The cognitive phenotype of this model has been conflicting. Working memory, assessed in the Y-maze has been shown to be impaired in some studies^3,5,6^, but not others^7–9^. Although an early study reported tone and contextual fear memory impairments from 6 months of age^10^, these results have not been replicated for contextual memory until much older ages^11,12^ and mixed results for tone fear^13,14^. Similarly, spatial memory in the water maze was reported as impaired as early as 4 to 6 months^6,10^ although others have reported intact memory up to 10 months^4,7^. The aversive stimuli used in the fear conditioning and the Morris water maze can induce stress^15^ which affect memory consolidation^16^. Appetitive tasks may help to overcome this limitation to test learning and memory in the *App*^*NL-G-F/NL-G-F*^ model.

Encoding and retrieving spatial information is a prominent everyday memory function that is affected in people living with AD^17^. This can be simulated in rodents with the appetitive delayed matching-to-place (ADMP) task in which food rewards are hidden in a specific location during the encoding trial and the animals’ ability to recall the location after a delay is indicative of their spatial memory^18,19^. Weak encoding in this task is shown to induce a short-lasting memory. However, novelty introduced in a close timeframe around the weak encoding leads to long-term memory^20–24^. Peri-learning novelty facilitates memory, likely through an underlying process called behavioural tagging and capture (BTC). The BTC hypothesis emphasises the importance of a strong event (e.g. novelty) in providing plasticity-related products which are captured by tags set by a learning event and in contributing to memory persistence of the learning event^25–27^. The BTC hypothesis was developed following the synaptic tagging and capture hypothesis which describes the underlying process in electrophysiology studies showing, in brief, that a separate strong stimulation enables lasting changes of plasticity that are induced by weak stimulation. It has been hypothesised that the weak stimulation leads to setting of tags at synapses which enable capture of plasticity-related proteins (PRP) that are produced by a separate strong stimulation that converges to an overlapping neuronal population, and as a consequence leading to lasting change of plasticity^20,26,28,29^. Novelty-induced promotion of memory has been seen in a myriad of behavioral tasks^30–32^. This ADMP task has also been successfully used to demonstrate an age-dependent memory decline in early ageing^23^ and a requirement of the locus coeruleus-hippocampal circuit for novelty-promoted memory^21^, brain regions which are affected in AD^33^.

Evidence above suggests sensitivity in detecting cognitive aging by the ADMP task and reveals circuits required for novelty-promoted memory. Hence, we used this ADMP task and asked how memory persistence and novelty-promoted memory are affected in this knock-in mouse model of AD at mature adulthood (around 6-month-old) and at middle age (around 12-month-old), as ageing is a major risk factor in AD^4,34^. We first compared *App*^*NL-G-F/NL-G-F*^ and wild-type littermates and asked if there was group difference in learning, in short-term memory (STM), and in long-term memory (LTM). We next asked if peri-learning novelty, via exposure to a novel box or novel objects after encoding, could improve memory persistence in both groups. Finally, we measured the animals’ performance in a Y-maze^3^, in an open field^7^, in a novel object recognition (NOR) task^7^, in a novel location recognition (NOL) task^35^, and in a Barnes maze^11^.

## Materials and methods

### Animals and experimental design

*App*^*NL-G-F/NL-G-F*^ mice (n = 13, with 5 being female) and wild-type (WT) littermates (n = 12, with 8 being female) were bred in-house from *App*^*NL-G-F/WT*^ breeding^3^. Mixed genotypes were group housed (2–5 mice/cage) and kept on a 12:12 h light:dark cycle (7:00–19:00 being the light phase during which the study was conducted). Food and water were provided ad libitum except during the ADMP task when the mice were food-restricted to 85–90% free-feeding body weight. They received a week of daily handling and habituation to transportation. They then underwent the ADMP, Y maze, open field, NOR, NOL tasks, followed by the Barnes maze task in adulthood (6–9 months) and then the ADMP, Y maze, NOR, and NOL tasks in middle age (12–14 months). The experimenters were blind to the animals’ genotype in behavioral training and scoring. All procedures were carried out under a Project Licence that was approved by the University of Edinburgh’s Animal Welfare and Ethical Review Body and UK Home Office. Experiments were additionally reviewed and approved by the Experimental Request Team at the Bioresearch and Veterinary Service of the University of Edinburgh, which provided and approved additional checks on animal welfare and ethics. All protocols were in accordance with the Home Office Animals Scientific Procedures Act 1986 (amended 2012). The study was designed according to the ARRIVE guidelines. All methods were carried out in accordance with relevant guidelines and regulations.

### Experimental apparatuses

#### ADMP arena

ADMP training took place in a square dry-land arena (90 × 90 × 35 cm) surrounded by clear Plexiglas walls allowing observation of visible cues outside of the maze. Entrance to the arena occurred via darkened start boxes (L/W/H: 21 × 14.5 × 15 cm) situated at the centre of each of the 4 walls. The arena floor was composed of a 5 × 5 Plexiglas grid covered by clean bedding. Two of the grid locations were occupied by the intra-arena cues (a black torch and a white tin located at columns 2 and 4 of row 3). In each session, 5 sandwell locations were set out in a map of 2 mid-distance and 3 far from the start box. Entry was rotated between the different start boxes every training day. Sandwells were filled with sand and contained an inaccessible bottom layer of 10 multigrain Cheerio rewards to mask odours. Correct sandwells were rewarded with ½ Cheerios (called pellets or rewards) within the sand (about 1.5 cm in depth) and a ¼ Cheerio in the start box (called cue pellet). Sessions were recorded via a webcam placed above the arena.

#### Open field, box, and objects

A Perspex box (40 × 40 × 40 cm) with clear plastic walls and floor was placed at the centre of the arena (with intra-arena cues removed). For peri-learning box novelty, novel substrates such as feathers or shredded paper were used to completely cover the floor of the box. For peri-learning object novelty, identical pairs of novel objects such as LED lightbulbs (D/H: 4.5 × 5 cm) or red post boxes (D/H: 3.2 × 8.5 cm) were placed in the box. For the open field, the floor was left clear with normal arena bedding visible below the Perspex floor. In the case of the NOL and NOR tasks, the floor was lightly covered in bedding. For the NOL task, a pair of transparent Pyrex jars with a blue lid (D/H: 5 × 10.5 cm) were used. For the NOR task, pairs of objects that did not induce innate between-object bias in mice were presented e.g., red phone boxes (L/W/H: 8.5 × 3 × 3 cm) and white 3-prong plugs (L/W/H: 5 × 5 × 7 cm).

#### Y-maze

The Y-maze comprised of 3 white opaque Plexiglas arms (L/W/H: 40 × 7 × 12 cm) positioned at 120° angles from each other with a 7 × 7 × 7 cm triangular centre area.

#### Barnes maze

The Barnes maze was a 91.5 cm diameter white plastic circle with 20 holes (D: 5 cm) placed 3 cm away from the edge of the maze. Beneath a designated escape hole, a black plastic box (L/W/H: 10.5 × 6 × 6 cm) could be inserted. The location of the escape hole was changed across animals and counterbalanced. An opaque white plastic cylinder (H/D: 23 × 15 cm) was placed in the centre of the circle as the starting point for each trial. Bright light (250–300 lx) and white noise (85 dB) were used as aversive stimuli to encourage the mice to search for and enter the escape box. Between animals, the maze was cleaned with 70% ethanol (30% for the escape box) to reduce odours.

### Behavioral tasks and procedures

#### The ADMP task

The ADMP task is an everyday spatial memory task that encourages the animals to encode a unique rewarded location on the day and update it across days. It was composed of habituation, initial training sessions, probe tests to assess memory, and interleaving training sessions^21^.

##### Habituation

To habituate the mice to dig for rewards, sandwells were placed in the home cage with pellets becoming gradually deeper in the sand over 4 sessions, then they were habituated to digging the sandwell in the centre of the arena. Mice were encouraged to return to the start box to eat the reward. Once 2 rewards were retrieved in less than 5 min, the mice could move on to the regular training schedule.

##### Training

Each training session consisted of 2 encoding trials and 1 retrieval trial per day separated by intervals of approximately 10 min each. In an encoding trial, one rewarded sandwell was placed in the arena. The mouse was then placed in the start box, consumed a small cue pellet, and entered the arena to search for the sandwell. Mice would return to the start box to consume the pellet before returning to the arena to retrieve the second pellet. The location of the rewarded sandwell was counterbalanced across days and genotypes. During the retrieval trial, one rewarded sandwell was placed at the location that matched to the encoding one and 4 unrewarded sandwells were also added in the arena. The trial was ended when the mouse collected both rewards. The latency (in seconds) to dig at the correct sandwell and the number of errors in digging at the unrewarded sandwells (max 4, min 0, chance 2) were recorded as indices of learning.

##### Memory probes

After the mice went through initial training, memory was assessed via post-encoding probe tests that consisted of 5 unrewarded sandwells. A 60 s timer was started following the first dig at any sandwell. Time spent at digging the correct (i.e. matching to the encoding location) and incorrect sandwells was recorded. The percentage of correct digging time over total digging time served as the index of memory and chance level was 20%. After the 60 s, the experimenter placed 2 rewards in the correct well to prevent extinction of digging. At least 1 training session interleaved probe tests to prevent extinction of the matching-to-place rule. STM was assessed in a probe test 10-min after 2 encoding trials of 2 pellets per trial. LTM was assessed in a probe test 24 h after 2 encoding trials with 4 pellets per trial (strong encoding) or after 1 encoding trial with 2 pellets (weak encoding). Intermediate-term memory (ITM) was assessed 6 h after 1 encoding trial with 2 pellets. Exploration in a novel box or with novel objects for 5 min may occurred 40 min after encoding to determine if peri-learning novelty improves memory persistence.

#### Open-field exploration

Mice were placed at the centre of the Perspex box for 5 min. Exploration was recorded via an overhead camera using Anymaze software (Stoelting Co., IL, USA). Time spent in the zones representing the inner 50% and outer 50% area of the box and the distance travelled (not including rearing) were measured. This 5-min trial also served as the first session of six habituations to the Perspex box for the novel object recognition and novel object location tasks.

#### The novel object recognition task

In the sampling phase, mice were given 10 min to explore 2 identical objects placed in opposite corners, 10 cm away from the Plexiglas box walls. In the 5-min test phase, 24 h after the sampling phase, one object had been replaced by a different object. The location of sampling and testing objects, and identity of objects pairs were counterbalanced across animals. Exploration of both objects was scored manually by the experimenter when the animals’ head pointed toward and came within 2 cm of the object^36,37^. A discrimination index was calculated using the following formula:

$$\frac{novel\, object\, exploration\, (s) - familiar\, object\, exploration\, (s)}{total\, object \,exploration\, (s)} \times 100$$

#### The novel object location task

In the sampling phase, the mice were given 10 min to explore two identical objects placed 10 cm away from the corners, against the same side of the box. In the 5-min test phase, 6 h after the sampling phase, one object was moved to the centre of the opposite wall and the animal was given 5 min to freely explore the new setup. A 6 h delay was used due to unreliable 24-h NOL memory using this sampling duration in a pilot study. Exploration of both objects was scored manually by the experimenter with the criteria describe above. The location of sampling and displaced objects was counterbalanced across animals. A discrimination index was calculated using the following formula:

$$\frac{displaced\, object\, exploration\, (s) - nondisplaced\, object\, exploration\, (s)}{total\, object\, exploration\, (s)}\times 100$$

#### The Y-maze task

The Y-maze task was carried out to assess the animals’ spontaneous tendency to alternate among the 3 arms of the maze, often linked to working memory^38,39^. Each mouse was placed at the centre of the maze with the facing arms counterbalanced between mice. The sequence of arm visits was recorded for 5 min. Alternation was defined as the mouse entering 3 different arms consecutively (chance: 22.2%). An alternation index (%) was calculated by using the formula^39,40^:

$$\frac{number \,of\, alternating\, arm\, entries}{total\, number\, of\, arm\, entries-2} \times 100.$$

#### The Barnes maze task

The mice were habituated to the start cylinder, the maze itself, and the escape box for 2 days prior to training. For each training trial, the mice were placed in the start cylinder at the centre of the maze for 10 s followed by exposure to the whole maze with loud noise and bright light. The trial ended when the mouse entered the escape box and was capped at 3 min when the experimenter guided the mouse to enter the escape box. Training consisted of 5 daily sessions of 2 trials in each session. The escape location was kept consistent per animal and counterbalanced across animals. In the STM and LTM probes, 1 and 24 h after training respectively, the mice had 60 s in the maze with white noise and bright light but without the escape box. Time spent in each quadrant was recorded and scored using Anymaze. Mean speed was scored by Anymaze as total distance travelled (m)/total time (sec). The escape box was replaced at the end of the probe to prevent extinction.

### Histology

Animals were anaesthetised deeply with pentobarbital and transcardially perfused with 1 × phosphate buffered saline (PBS) then 4% paraformaldehyde around 14 months old. Coronal brains sections (40 µm) were collected. To stain for β-Amyloid, sections were blocked in 10% normal goat serum with 0.1% Triton™ X-100 (Sigma Aldrich) in PBS for 1 h followed by an overnight incubation in primary antibody (1:500 mouse anti-β-Amyloid, 6E10, BioLegend). Sections were incubated in the secondary antibody for 2 h (1:100 anti-mouse Biotin, Sigma Aldrich), followed by the Avidin–Biotin complex (ABC elite, standard, Vectastain), and 3.3′-diaminobenzine (DAB, Vector Laboratories). Sections were mounted on glass slides and coverslipped with dibutylphthalate polystyrene xylene (DPX, Sigma Aldrich). Note, sections from one *App*^*NL-G-F/NL-G-F*^ mouse were excluded due to hippocampal tissue damage in processing. Hippocampal images were acquired via Zeiss, Axio Scan.Z1 (200 micro-sec, 1.72 µm depth of focus). Images were analysed using ImageJ (1.52n) to measure the percentage area with positive 6E10 signal and manual plaque number counting.

### Statistics

All statistical analysis was done using SPSS Statistics 24 (IBM). Two-way mixed ANOVAs with sphericity checks were used for reporting 2 factors and interactions, such as training and genotype effects in training errors and latencies. Genotype comparisons in designs with a single factor were run using independent samples *t* tests and checked for equal variance. If the data did not fulfil normality, a Mann–Whitney *U* test was run. Comparisons against a fixed value (e.g. chance or 0) were checked for normality and then analysed by 1-sample *t* tests or a related-samples Wilcoxon signed rank test if normality failed.

## Results

### *App*^*NL-G-F/NL-G-F*^, compared to wildtype, mice do not display learning or memory impairments in the ADMP task

**Figure 1:**
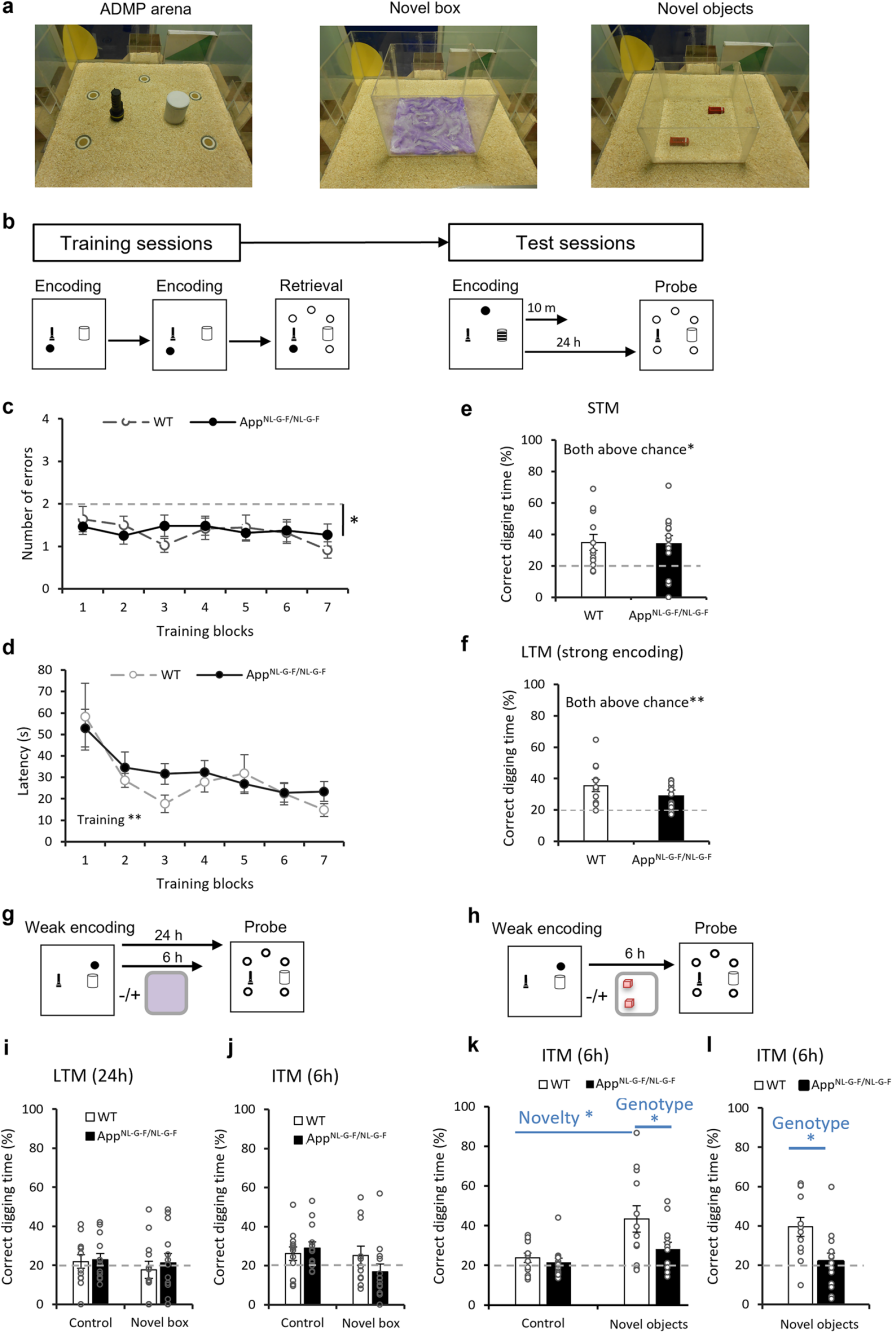
Appetitive spatial learning, memory, and novelty-promoted memory (via BTC) in adulthood. (**a**) The event arena (left), a box with novel substrates (middle), and a box with novel objects (right). (**b**) The ADMP training and testing procedures. (**c**) Comparable task acquisition in WT and App^NL-G-F/NL-G-F^ mice across 7 training blocks. The errors were significantly below chance. (**d**) Comparable efficiency (latencies in the retrieval trials) in WT and App^NL-G-F/NL-G-F^ mice across 7 training blocks and the reduction over training was significant. (**e**) STM and (**f**) LTM were comparable between genotypes and significantly above chance. (**g**–**j**) The novelty-promoted memory comprised of a weak encoding, followed 40 min later by exploration in a box with novel substrates. LTM and ITM were not significantly above chance in both groups. (**h**–**l**) The novelty-promoted memory comprised of a weak encoding, followed 40 min later by exploration in a box with novel objects. The percentage of time digging at the correct location was significantly above chance for WT, but not App^NL-G-F/NL-G-F^ mice after exploration of novel objects (**k**) and in replication (**l**). Data are presented as mean ± SEM. *p < 0.05, **p < 0.01.

A series of behavioral tasks were performed, starting with the ADMP task (Fig. 1a,b). Acquisition of the ADMP task in adulthood was first assessed by the number of errors made during the retrieval trial (Fig. 1c). It was averaged over 7 blocks of 3 training sessions per block and a 2-way ANOVA was performed to assess the training effect, genotype effect and the interaction of the two (Fig. 1c). Both genotypes made a similar number of errors during training (F_1,23_ = 0.18, p = 0.7). The effect of training block and the interaction were both insignificant (F_6,138_ = 0.76, p = 0.6 and F_6,138_ = 0.65, p = 0.7, respectively). Importantly, the errors made during the 7 blocks of training were significantly below chance (block 1 to block 7: t_24_ = − 2.71 to − 5.51, p < 0.02 to p < 0.0001), indicating the mice learnt the task early on and persistently performed well.

The latency to find the reward during the retrieval trials revealed a highly significant training effect (Fig. 1d, F_2.9,66.9_ = 6.45, p = 0.001) and the linear trend was also significant (F_1,23_ = 14.94, p = 0.001). This suggests that mice improved in task efficiency over time. The genotype effect and interaction were both insignificant (F_1,23_ = 0.76, p = 0.4 and F_2.9,66.9_ = 0.56, p = 0.6), suggesting a similar rate of improvement in both groups.

In the STM test, mice showed a significant preference for digging in the previously rewarded (i.e. correct) sandwell compared to chance (Fig. 1e, WT: t_11_ = 2.97, p = 0.013, and *App*^*NL-G-F/NL-G-F*^: t_12_ = 2.60, p = 0.023), with no difference between the two genotypes (t_23_ = 0.15, p = 0.9). In the LTM test, mice showed a significant preference for digging in the correct sandwell (Fig. 1f, WT: t_11_ = 4.10, p = 0.002, *App*^*NL-G-F/NL-G-F*^: t_12_ = 4.06, p = 0.002). There was no significant effect of genotype (Mann–Whitney U = 53, p = 0.2). Together, these suggest that the *App*^*NL-G-F/NL-G-F*^ mice learnt and remembered the location similarly to their WT littermates.

### *App*^*NL-G-F/NL-G-F*^ mice show impairment in novelty-promoted memory

Next, we investigated the effect of peri-encoding novelty in memory persistence. After one weak encoding trial with 2 pellets^21^, mice explored a box with novel substrates for 5-min or remained in the home cage. After a retention period of 24 or 6 h after encoding, a probe test was conducted to assess LTM or ITM (Fig. 1g). Weak encoding alone was insufficient to induce LTM as both genotypes performed indifferent from chance (Fig. 1i left, WT: t_11_ = 0.55, p = 0.6, *App*^*NL-G-F/NL-G-F*^: t_12_ = 0.97, p = 0.4, insignificant group difference: t_23_ = − 0.23, p = 0.8). Peri-encoding novel box exploration was insufficient to induce LTM in either genotype (Fig. 1i right, WT: t_11_ = − 0.51, p = 0.6, *App*^*NL-G-F/NL-G-F*^: t_12_ = 0.26, p = 0.8, insignificant group difference: t_23_ = − 0.54, p = 0.6). Main effects of novelty, genotype, and interaction were all insignificant (novelty: F_1,23_ = 0.56, p = 0.5, genotype: F_1,23_ = 0.31, p = 0.6, interaction: F_1,23_ = 0.10, p = 0.8). After reducing the retention delay to 6 h (ITM), main effects of novelty, genotype, and interaction remained insignificant (Fig. 1j, novelty: F_1,22_ = 3.18, p = 0.1, genotype: F_1,22_ = 0.37, p = 0.6, interaction: F_1,23_ = 2.57, p = 0.1).

Novelty through exploration of novel objects after encoding was introduced (Fig. 1h). The results showed that encoding followed by novel object exploration, compared to encoding alone, led to ITM in the WT mice (Fig. 1k, t_11_ = 2.69, p = 0.021). The genotype effect was significant (Fig. 1k, F_1,23_ = 4.37, p = 0.048) although the interaction only trended towards significance (F_1_,_23_ = 3.02, p = 0.095). A replication of this procedure with a different set of novel objects further supported this genotype difference (Fig. 1l, genotype: t_23_ = 2.76, p = 0.011). Together, these results would suggest an impairment of peri-learning novelty in promoting memory in the AD mice.

### Ageing effect in mice in the ADMP task

**Figure 2:**
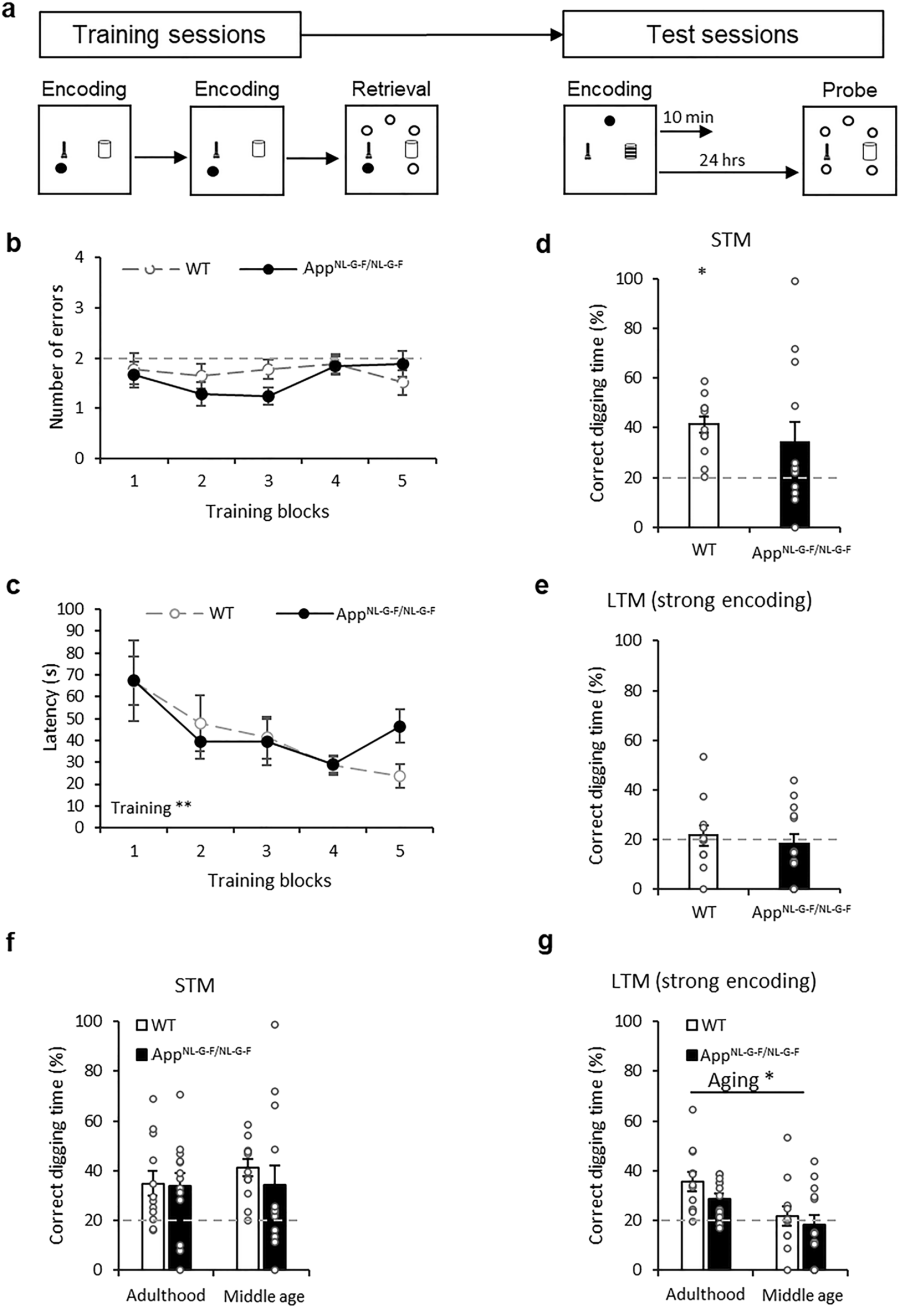
Appetitive spatial learning and memory in middle age. (**a**) The ADMP training and testing procedures. (**b**) Errors during 5 blocks of training were not significantly below chance. (**c**) Latencies were significantly reduced over the 5 training blocks (**d**) STM was comparable between groups. (**e**) LTM was comparable between groups but not significantly above chance. (**f**) No age-dependent decline in STM. (**g**) Age-dependent decline in LTM. Data are presented as mean ± SEM. *p < 0.05.

It has been reported that rats can retain their training performance from adulthood to middle-age with a long gap of no training^23^. Hence, we asked whether mice could maintain their performance over time (Fig. 2a). At 12-month-old, errors did not decrease over training blocks (Fig. 2b, F_4,92_ = 0.94, p = 0.4). The genotype effect and interaction were insignificant (F_1,23_ = 1.10, p = 0.3; F_4,92_ = 1.03, p = 0.4, respectively). Critically, errors in the first block were not significantly below chance (Fig. 2b, t_24_ = − 1.39, p = 0.2). This would suggest that the error-based performance did not maintain from adulthood to middle age.

At middle age, the latency to retrieve the reward reduced across training blocks (Fig. 2c, F_3,64_ = 4.60, p = 0.007; a significant linear trend, F_1,23_ = 12.39, p = 0.002). Genotype and interaction effects were insignificant (F_1,23_ = 0.13, p = 0.7; F_3,65_ = 0.76, p = 0.5, respectively). Critically, the latency at the first block at 12-month-old was significantly higher than the latency at the last block at adulthood (Paired samples Wilcoxon Signed Ranks p < 0.001). This would suggest that the task efficiency does not maintain to an older age although it improves after further training. While there was not a significant genotype effect in efficiency overall, AD mice were less efficient in the last training block (Fig. 2c, block 5, Mann–Whitney U = 29, p = 0.007).

Memory assessment at middle age revealed the ability to form and express STM was maintained as correct digging percentages were significantly above chance in WT mice (Fig. 2d, t_11_ = 5.90, p < 0.001, not for *App*^*NL-G-F/NL-G-F*^: t_12_ = 1.79, p = 0.1 due to variation). The genotype effect was insignificant (t_16.2_ = 0.66, p = 0.5). LTM in middle-aged mice was not apparent (Fig. 2e, both groups not above chance, WT: t_11_ = 0.41, p = 0.7, *App*^*NL-G-F/NL-G-F*^: t_12_ = − 0.45, p = 0.7). The genotype effect was not significant (t_23_ = 0.60, p = 0.6).

STM was sustained in adulthood and in middle age (Fig. 2f; age: F_1,23_ = 0.30, p = 0.6; genotype: F_1,23_ = 0.28, p = 0.6; interaction: F_1,23_ = 0.21, p = 0.7). However, middle-aged animals showed impaired LTM (Fig. 2g; age: F_1,23_ = 10.71, p = 0.003). Both genotype and interaction were insignificant (F_1,23_ = 2.24, p = 0.2; F_1,23_ = 0.19, p = 0.7, respectively). Together, the ADMP task reveals an early cognitive aging effect in LTM.

### Intact memory in the Barnes maze in *App*^*NL-G-F/NL-G-F*^ mice, although they take longer to reach the target

**Figure 3:**
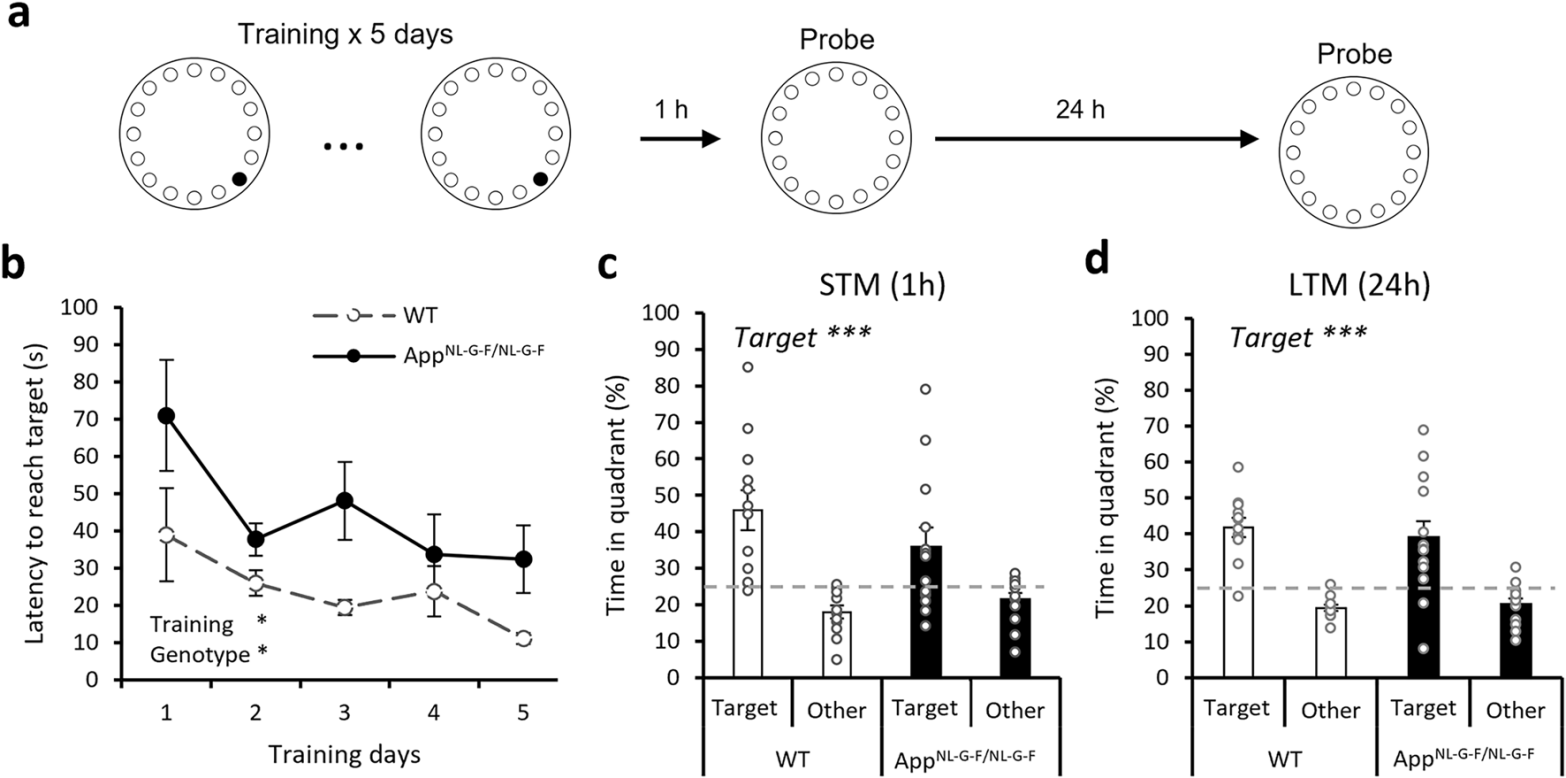
Aversive spatial learning and memory in the Barnes maze. (**a**) The training and probe test procedures. (**b**) The latencies were significantly reduced over 5 training days. The genotype effect, but not the interaction, was significant. (**c**) STM in both groups was indicated by significantly more time spent in the target quadrant than averagely in other quadrants (p < 0.001) and this was not affected by the genotype. (**d**) LTM in both groups was indicated by significantly more time spent in the target quadrant than averagely in other quadrants (p < 0.001) and this was not affected by the genotype. All data presented as mean ± SEM. *p < 0.05, ***p < 0.001.

The Barnes maze task involved the use of aversive stimuli to motivate animals to locate an escape box. Learning was demonstrated by decreasing latencies to reach the escape box across training sessions (Fig. 3a,b, training session: F_2.4,55_ = 5.29, p = 0.005). The interaction was insignificant (F_2.4,55_ = 0.83, p = 0.5), which suggests that both groups acquired the task at a similar rate. However, *App*^*NL-G-F/NL-G-F*^ mice took significantly longer to reach the escape box (F_1,23_ = 6.46, p = 0.018). This was not due to genotypic differences in motor ability as speed did not differ between the two genotypes (F_1,23_ < 0.1, p = 1) and their speed increased over training sessions (F_4,92_ = 3.78, p = 0.007) with no significant group by session interaction (F_4,92_ = 1.60, p = 0.2).

In the STM probe, there was no significant genotype effect (Fig. 3c, F_1,23_ = 1.73, p = 0.2). The time spent in the target quadrant was significantly higher than in the other quadrants averaged (F_1,23_ = 17.14, p < 0.001). The genotype–quadrant interaction was not significant (F_1,23_ = 1.74, p = 0.2). In the LTM probe, there was also no significant genotype difference (Fig. 3d, F_1,23_ = 0.287, p = 0.597). The time spent in the target quadrant was also significantly higher than in the other quadrants averaged (F_1,23_ = 31.23, p < 0.001). The genotype–quadrant interaction was not significant (F_1,23_ = 0.285, p = 0.599). Thus, the Barnes maze task did not reveal a robust genotype effect on STM or LTM.

### Intact open field exploration, spontaneous alternation, and recognition memory in *App*^*NL-G-F/NL-G-F*^ mice

**Figure 4:**
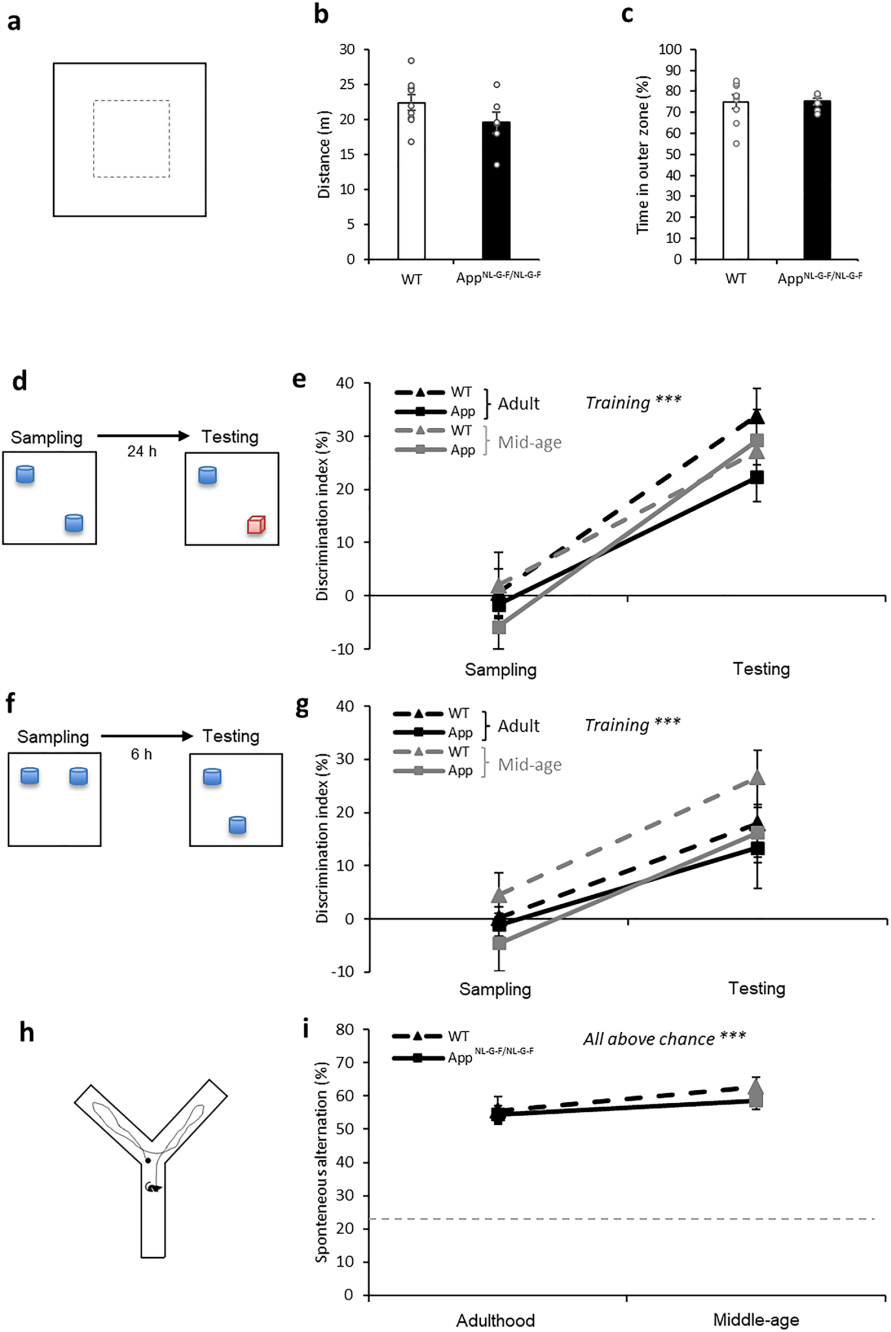
Behavioral results for the open field, novel object recognition (NOR), novel object location (NOL) tasks, and the Y-maze. (**a**) A schematic of the open field. (**b**) Distance travelled was comparable between groups in adulthood. (**c**) Time spent in the outer zone of the box was comparable between groups in adulthood. (**d**) A schematic of the NOR task. (**e**) Learning of NOR led to significant increase in discrimination index from training to testing (p < 0.0001), while the genotype effect, aging effect, and interactions were not significant. (**f**) A schematic of the NOL task. (**g**) Learning of NOL recognition led to significant increase in discrimination index from training to testing (p < 0.0001), while the genotype effect, aging effect, and interactions were not significant. (**h**) A schematic of the Y-maze. (**i**) Spontaneous alternation in the Y-maze was not affected by genotype. It was significantly above chance in both groups and at both ages (all p < 0.001). All data presented as mean ± SEM. ***p < 0.001.

Spontaneous exploratory activity can be assessed by distance travelled within the open field task. This was not significantly different between *App*^*NL-G-F/NL-G-F*^ mice and their WT littermates (Fig. 4a,b; t_13_ = 1.52, p = 0.2). Thigmotaxis in the open field task can be used to measure anxiety-behavior^38^. *App*^*NL-G-F/NL-G-F*^ mice did not show significant differences compared to their WT littermates in time spent in the outer zone (Fig. 4c; t_13_ = − 0.04, p = 0.9). Thus, these results show that exploration and anxiety in an open field are normal in the *App*^*NL-G-F/NL-G-F*^ mice.

For novel object recognition, we asked if the animals learned and remembered the task at the test in contrast to the performance in sampling (Fig. 4d), if there was a group difference based on the genotype, and if aging from adulthood to the middle age affects recognition. To answer these, we performed a 3-way ANOVA on the data. The animals showed obvious learning and remembering, which effect was supported by a significant increase in the discrimination index from sampling to testing (Fig. 4e, F_1,23_ = 85.83, p < 0.0001). The genotype effect was not significant (F_1,23_ = 1.8, p = 0.2). The aging effect (F_1,23_ = 0.04, p = 0.8) was not significant. All 2-way and 3-way interactions were not significant (all F_1,23_ = 0.002–1.91, all p = 0.2–1.0). We verified that the performance at the baseline discrimination at sampling was not significantly different from chance (Fig. 4e, *t* tests against zero, WT: p = 0.7, App^NL-G-F/NL-G-F^: p = 0.2) and the performance at testing was significantly above chance (*t* tests against zero, both groups with p < 0.001). These results would suggest that the performance in the NOR task and the degree of learning in the App^NL-G-F/NL-G-F^ mice remain comparable with those in the WT mice.

Similar analytical approaches were applied to data from the novel object location task (Fig. 4f). The animals showed obvious learning and remembering, which effect was supported by a significant increase in the discrimination index from sampling to testing (Fig. 4g, F_1,23_ = 23.87, p < 0.0001). The genotype effect was not significant (F_1,23_ = 2.86, p = 0.1). The aging effect (F_1,23_ = 0.5, p = 0.5) was not significant. All 2-way and 3-way interactions were not significant (all F_1,23_ = 0.02–0.61, all p = 0.4–0.9). We verified that the performance at the baseline discrimination at sampling was not significantly different from chance (Fig. 4g, *t* tests against zero, WT: p = 0.4, App^NL-G-F/NL-G-F^: p = 0.4) and the performance at testing was significantly above chance (*t* tests against zero, both p < 0.01). These results would suggest that the performance in the NOL task and the degree of learning in the App^NL-G-F/NL-G-F^ mice remain comparable with those in the WT mice.

Studies using the spontaneous Y-maze task to assess spatial working memory have reported mixed results at 6 months of age in this model^3,7^. We therefore investigated this function as well. The alternation percentage (Fig. 4h,i) was not significantly affected by genotype (F_1,23_ = 0.62, p = 0.4) or by genotype–aging interaction (F_1,23_ = 0.34, p = 0.6), and mildly by aging (F_1,23_ = 4.50, p = 0.045). Both genotypes’ alternation was significantly above chance in adulthood (WT: t_11_ = 8.24, p < 0.0001, *App*^*NL-G-F/NL-G-F*^: t_12_ = 13.31, p < 0.0001) and in middle-age (WT: t_11_ = 14.36, p < 0.0001, *App*^*NL-G-F/NL-G-F*^: t_12_ = 13.81, p < 0.0001). Thus, *App*^*NL-G-F/NL-G-F*^ mice do not show impairment in spontaneous alternation.

### Amyloid load at middle age

**Figure 5:**
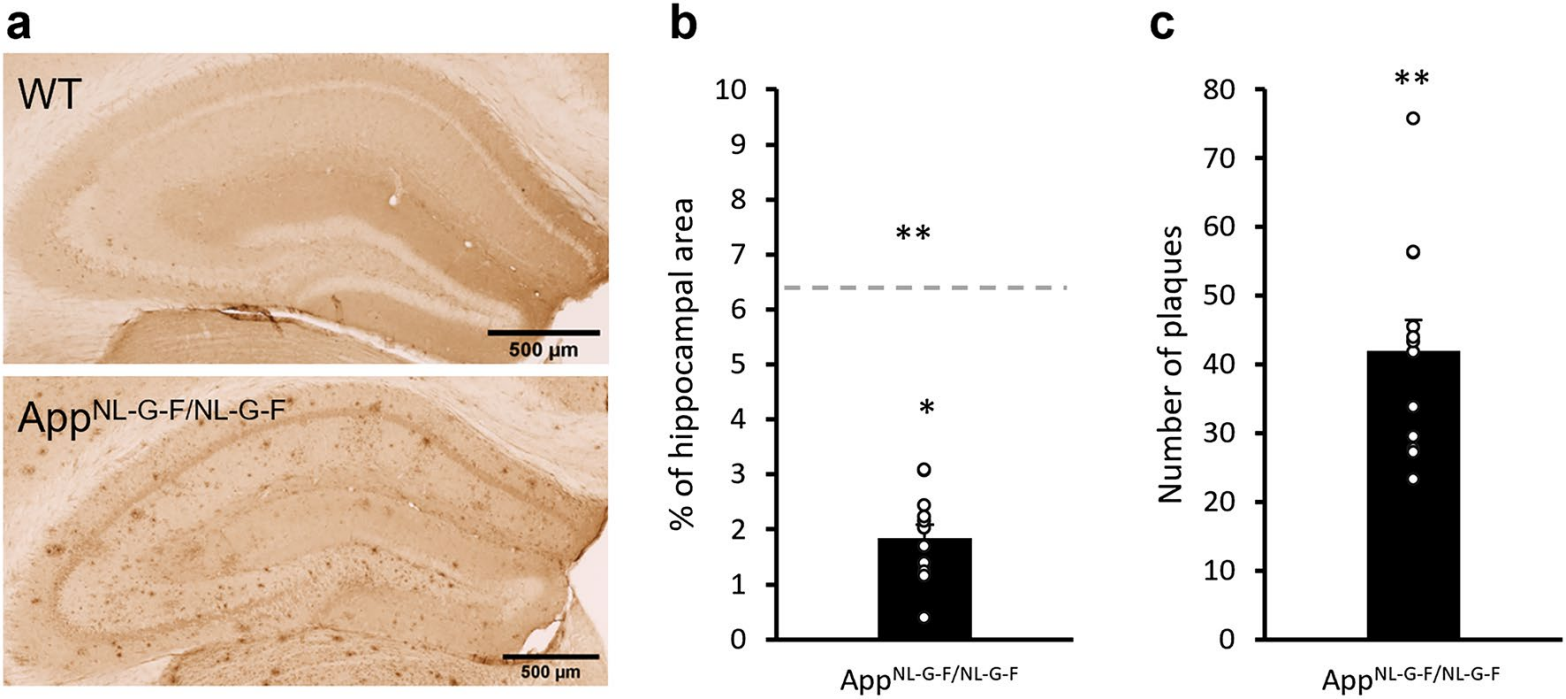
Immunohistochemistry (6E10) for showing amyloid burden. (**a**) Representative hippocampal images of a WT mouse (top) and a App^NL-G-F/NL-G-F^ mouse (bottom). (**b**) The percentage of 6E10-positive hippocampal area was significantly above 0 but below what was reported (dashed line inferred from Saito et al.^3^). (**c**) The number of plaques in the hippocampus was significantly above 0. Data is presented as mean ± SEM. *p < 0.05, **p < 0.01.

Since genotype difference in memory impairment was mainly seen in behavioral tagging but not other measurements even at middle age, we set out to determine if the plaque burden in the hippocampus was similar to what had been previously reported. At the end point around 14 months old, *App*^*NL-G-F/NL-G-F*^ mice showed clear amyloid pathology. In the hippocampus, the area covered in amyloid plaques was 1.8 ± 0.2% (Fig. 5a,b), which was significantly higher than 0 (t_11_ = 7.98, p < 0.0001), but significantly lower than previously reported (t_14_ = − 10.51, p < 0.0001, vs. 6.4% in 9-months old *App*^*NL-G-F/NL-G-F*^ mice in Saito et al*.*^3^). The number of plaques in the hippocampus was also significantly higher than 0 (Fig. 5c, 42.0 ± 4.4, t_11_ = 9.56, p < 0.0001).

## Discussion

In this study, adult *App*^*NL-G-F/NL-G-F*^ mice show impaired novelty-promoted memory (via BTC) in the ADMP task while STM and LTM in this task, STM and LTM in the Barnes maze, LTM in NOR, and ITM in NOL remain intact (Table 1). This ADMP task also sensitively reveals age-dependent decline in LTM in early aging. The intact LTM in adult *App*^*NL-G-F/NL-G-F*^ mice may suggest that strong encoding would lead to sufficient tagging and PRPs in the activated cell populations in the hippocampus to sustain plasticity^29,41^ or structure changes^42–44^ for memory, despite amyloid pathology in the hippocampus at this stage^3,10,45^.

**Table 1:**
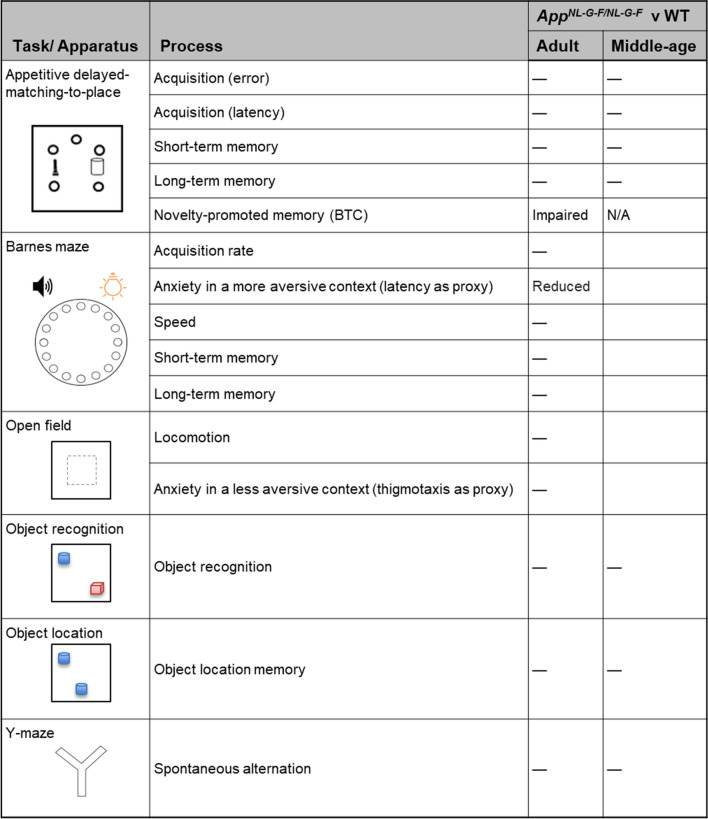
Summary of the behavioural tasks and results of the App^NL-G-F/NL-G-F^ compared to their WT littermates. *BTC* behavioural tagging and capture.

Peri-encoding novelty facilitates memory persistence in WT mice, but not in *App*^*NL-G-F/NL-G-F*^ mice. Using an overexpression model, APP/PS1 mice show weaker inhibitory avoidance learning compared to WT and impaired memory association through a BTC paradigm^46^. Here, we are able to rule out confounding from learning as *App*^*NL-G-F/NL-G-F*^ mice show comparable learning abilities with WT mice in this ADMP task and the findings suggest that the impairment in novelty-promoted memory precedes apparent learning or memory decline. It has been proposed that BTC relies on synaptic meta-plasticity processes and that memories persist when learning events set tags and events providing PRPs occur in spatial and in temporal proximity^25^. One underlying mechanism in causing the impairment in novelty-promoted memory in AD mice could be due to a decrease in the PRP synthesis.

Novelty-promoted memory in WT animals in this study (Fig. 1k,l) and in an earlier aging study (see Fig. 3E in Gros and Wang^23^) refers to an intermediate delay between encoding and testing. The intermediate-term memory has been shown to require protein synthesis^47,48^, although this has not been explicitly tested in this study. Whether object novelty promotes memory persistence at a longer delay (e.g. 24 h) in mice requires future investigation. The term ‘memory persistence’ can refer to LTM with a 24-h delay in the context of characterising the underlying receptor or molecular mechanisms on memory consolidation^49,50^. For example, it has been shown that protein synthesis is required for LTM, but not STM^51,52^, and for post-reactivation LTM but not post-reactivation STM^53,54^. Novelty is often introduced within a short time window around encoding or reactivation to promote memory persistence^20,22,30^. When looking at a longer-term memory at 7 days, Tomaiuolo et al. show that exploration in a novel open field at 11 h, but not 5, 8, or 24 h after learning can promote 7-day memory in inhibitory avoidance^55^. Whether this type of delayed novelty can promote longer-term memory in the ADMP task in a reactivation-independent manner^55^ or in a reactivation-dependent manner^22,56^ requires future research.

Novelty modulates neuronal activities in the locus coeruleus and ventral tegmental area, which send dopaminergic projections to the hippocampus^21^. Blocking dopaminergic receptors in the hippocampus prevents peri-encoding novelty from facilitating memory persistence^20,30^. Critically, blocking dopaminergic receptors in the hippocampus also prevents locus coeruleus stimulation from facilitating memory persistence^21^. Collective evidence leads to the view that locus coeruleus and ventral tegmental area send projections to the hippocampus which enables novelty detection^46,57^ and that such cross-region projections enable novelty-facilitated memory persistence through BTC^21,58^. Amyloid pathology is apparent in these midbrain structures in AD mouse models^59,60^ and in *App*^*NL-G-F/NL-G-F*^ mice^10,61,62^. Loss of dopaminergic neurons in midbrain is seen in familial AD mouse models with alpha-synuclein pathology^63^ or presenilin mutations^60^. Reduced locus coeruleus volumes are reported in people with mild cognitive impairment or dementia due to AD^64^ and brainstem volume reduction and deformation in people with clinical AD^65,66^. Together, it is likely that the circuits between midbrain and hippocampus are already affected at a younger age of AD mice^62^ which contribute to impaired novelty-promoted memory (via BTC).

At the cellular level, we have recently shown that encoding of the ADMP task and novelty engage overlapping cell populations in CA1 and CA3 of the hippocampus^24^. Further analyses reveal that novelty, compared to familiarity, preferentially activates more cells in the distal CA1 and increases the overlapping cell population in distal CA1 and proximal CA3^24^. The proximal CA3–distal CA1 regions and connection have been implicated in object novelty or recognition^67,68^. It is possible that exposure to novel objects activates this network more so than exploring a novel context or substrates. Longer exposure to a novel context or substrates beyond what we did in this study may be required to facilitate memory persistence in mice^32,69,70^. As peri-encoding novelty does not improve memory persistence in *App*^*NL-G-F/NL-G-F*^ mice, future investigation in the local CA3–CA1 circuit can verify if AD causes alterations of this circuit and leads to impairment in novelty-promoted memory^71,72^. Disruptions to glutamate transmission at the CA3–CA1 synapses have been observed following pathology onset in the *App*^*NL-G-F/NL-G-F*^ mice^73^. Indirect evidence hints toward distal, compared to proximal, CA1 being more vulnerable in showing pathology in early AD^74^.

Aging-dependent decline in LTM is sensitively revealed with the ADMP task. This impairment in middle age is consistent with what has been shown in a cross-sectional study^24^ and in a longitudinal study^23^ in rats, both suggesting an encoding impairment in early aging. While encoding would also be affected by aging in the *App*^*NL-G-F/NL-G-F*^ mice, the impairment in novelty-promoted memory at a younger age would suggest that early amyloid pathology may already affect production of PRP^29,75^ and/or the circuits mentioned above for novelty detection in contributing to PRP.

Our open field results are consistent with previous studies that show comparable performance in wildtype and *App*^*NL-G-F/NL-G-F*^ mice^7,10^, which suggests that *App*^*NL-G-F/NL-G-F*^ mice are not more anxious. While both STM and LTM are comparable between groups in the Barnes maze task, *App*^*NL-G-F/NL-G-F*^ mice take longer to reach the target. As they improve across training sessions, this does not reflect a learning deficit. The walking speed is also comparable between groups, suggesting no motor impairment. One potential explanation is that they are less anxious than wildtype mice in an environment with stronger aversive stimuli. Consistently, *App*^*NL-G-F/NL-G-F*^ mice spend more time in the open arms in an elevated plus maze, suggesting reduced anxiety in this model^4^. Whether an anxiogenic or anxiolytic profile is observed may depend on the age of the animal, the progress of pathology, the behavioral history of the animals, and the magnitude of anxiogenic stimuli^76^.

Consistent with previous NOR studies, *App*^*NL-G-F/NL-G-F*^ mice performed similar to wildtype mice^7,10^. Similar to NOR, NOL in our study fails to serve as a sensitive behavioral marker for AD phenotypes in App^NL-G-F/NL-G-F^ mice. Our results of spontaneous alternation in the Y-maze are in agreement with an earlier study^7^. While another study showed poorer alternation in the *App*^*NL-G-F/NL-G-F*^ mice, it is important to note that those *App*^*NL-G-F/NL-G-F*^ mice still performed significantly above chance^3^. The subtle differences across studies could reflect environmental factors, such as experimenters’ gender and cage enrichment which can affect animal stress levels^7,77,78^.

Notably, by middle-age our *App*^*NL-G-F/NL-G-F*^ mice had significantly lower hippocampal amyloid plaque load than reported at 9 months^3^. The longitudinal design of the study with repeated tasks may have had a protective effect on the development of the pathology as repeated spatial training^79^, treadmill running^80^ and environmental enrichment^81^ have all been shown to reduce amyloid levels in AD models.

## Conclusion

The *App*^*NL-G-F/NL-G-F*^ mouse model provides a new opportunity to revisit the effects of amyloid on cognition without APP overexpression. The results show impairment in novelty-promoted memory (via BTC) prior to significant deficits in learning or memory in these mice. The circuit mechanisms, such as midbrain-hippocampal projections and CA3–CA1 circuits, underlying this type of early impairment with amyloid pathology requires future investigation. In conjunction with other studies highlighting synaptic tagging and capture impairments before apparent cognitive decline^75,82^, this could provide a new diagnostic direction for early detection of AD-related functional impairment.

## Data Availability

The datasets generated and analysed during the current study are available from the corresponding author on request.

## Abbreviations

AD: Alzheimer’s disease
ADMP: Appetitive delayed matching-to-place
APP: Amyloid precursor protein
BTC: Behavioral tagging and capture
ITM: Intermediate-term memory
LTM: Long-term memory
NOR: Novel object recognition
NOL: Novel object location
PRP: Plasticity-related protein
STM: Short-term memory
WT: Wild-type
